# Equitable Access to Mental Health Services in Europe

**DOI:** 10.1192/j.eurpsy.2025.1654

**Published:** 2025-08-26

**Authors:** M. A. Rodrigues

**Affiliations:** 1 Universidade Anhembi Morumbi, São Paulo, Brazil

## Abstract

**Introduction:**

Equitable access to mental health services is essential for the well-being of European citizens. Despite advancements in health policies, significant disparities in access to care persist. Socioeconomic, geographical, cultural, and institutional factors contribute to these inequalities, disproportionately affecting vulnerable groups.

**Objectives:**

To analyze the inequalities in access to mental health services in Europe and identify effective strategies to promote equity in these services.

**Methods:**

This study conducted a systematic literature review using PubMed, Scopus, and PsycINFO databases to identify studies published between 2015 and 2023. The keywords used were “mental health access,” “health equity,” “Europe,” and “mental health inequalities.” Inclusion criteria focused on studies addressing access to mental health services in Europe and strategies to promote equity. Extracted data were qualitatively synthesized, highlighting trends, barriers, and facilitators to equitable access to mental health services.

**Results:**

The review revealed significant inequalities in access to mental health services in Europe, primarily due to socioeconomic and geographical factors. Low-income individuals face major barriers, such as high costs and lack of coverage by health systems. Rural areas suffer from a shortage of specialized professionals and inadequate infrastructure, making continuous quality care challenging.

Cultural and linguistic factors add further complexity, particularly for migrants and ethnic minorities. The lack of culturally adapted services and sensitivity in therapeutic approaches often leads to inadequate care. Stigma surrounding mental health remains a significant barrier, preventing many from seeking help.

Variations in mental health policies and investments across European countries further exacerbate these inequalities, creating a fragmented landscape of care access. While some countries have robust strategies, others lack consistent policies and adequate funding. Addressing these disparities requires integrated policies, culturally sensitive professional training, and effective stigma-reduction programs.

**Conclusions:**

There is an urgent need to address inequalities in access to mental health services in Europe. Integrated strategies are essential, including policies that promote equity, improvements in rural infrastructure, and culturally sensitive professional training. Reducing stigma through awareness programs is crucial to encourage help-seeking behavior. Harmonizing policies and investments among European countries can contribute to more equitable and effective access, ensuring equal opportunities for mental health treatment and support.

**Disclosure of Interest:**

None Declared

